# Supplementary material for: IGHMBP2 deletion suppresses translation and activates the integrated stress response
Source: bioRxiv. 2023 Dec 12:2023.12.11.571166. Preprint. [Version 2] doi: 10.1101/2023.12.11.571166 (PMC10760061; doi:10.1101/2023.12.11.571166)
Supplement: 3 [file NIHPP2023.12.11.571166V2-supplement-1.pdf]

bioRxiv preprint doi: https://doi.org/10.1101/2023.12.11.571166; this version posted December 12, 2023. The copyright holder for this preprint (which was not certified by peer review) is the author/funder, who has granted bioRxiv a license to display the preprint in perpetuity. It is made available under aCC-BY 4.0 International license.

Park_FigS1

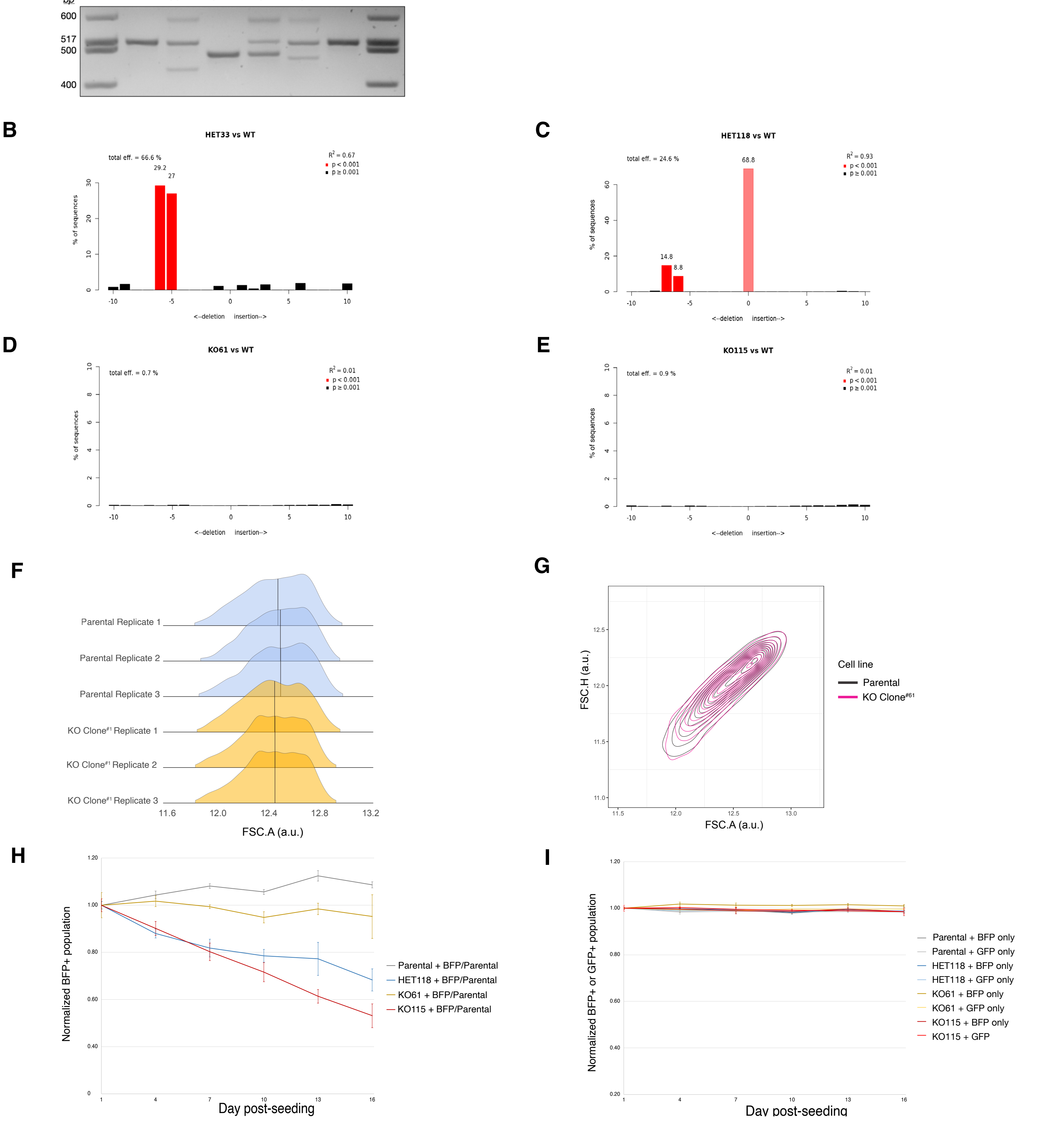

Figure S1. IGHMBP2 deletion clone genotyping and characterization.
(*A*) Genotyping of select clones via PCR around IGHMBP2 Exon 2 cut-site. (*B-E*) TIDE alignment of Sanger sequencing results quantifying indel frequencies among alleles. (*F*) Forward-scatter area distribution between representative IGHMBP2 KO Clone#1 and parental cells in arbitrary units. (*G*) Forward-scatter area versus height profiles between representative IGHMBP2 KO Clone#1 and parental cells. (*H*) Competitive proliferation profiles between ΔIGHMBP2 cell lines stably expressing BFP seeded with 50% non-fluorescent parental cells. Each sample was seeded in triplicate on Day 0 and independently passaged on each day of measurement. (*I*) Measurement of BFP+ population over 2 weeks in cell lines expressing transgenic BFP or GFP. For *G* and *H*, error bars reflect the standard deviation of BFP+ populations normalized to the mean Day 1 reading among triplicate wells per sample

bioRxiv preprint doi: https://doi.org/10.1101/2023.12.11.571166; this version posted December 12, 2023. The copyright holder for this preprint (which was not certified by peer review) is the author/funder, who has granted bioRxiv a license to display the preprint in perpetuity. It is made available under aCC-BY 4.0 International license.

Park_FigS2

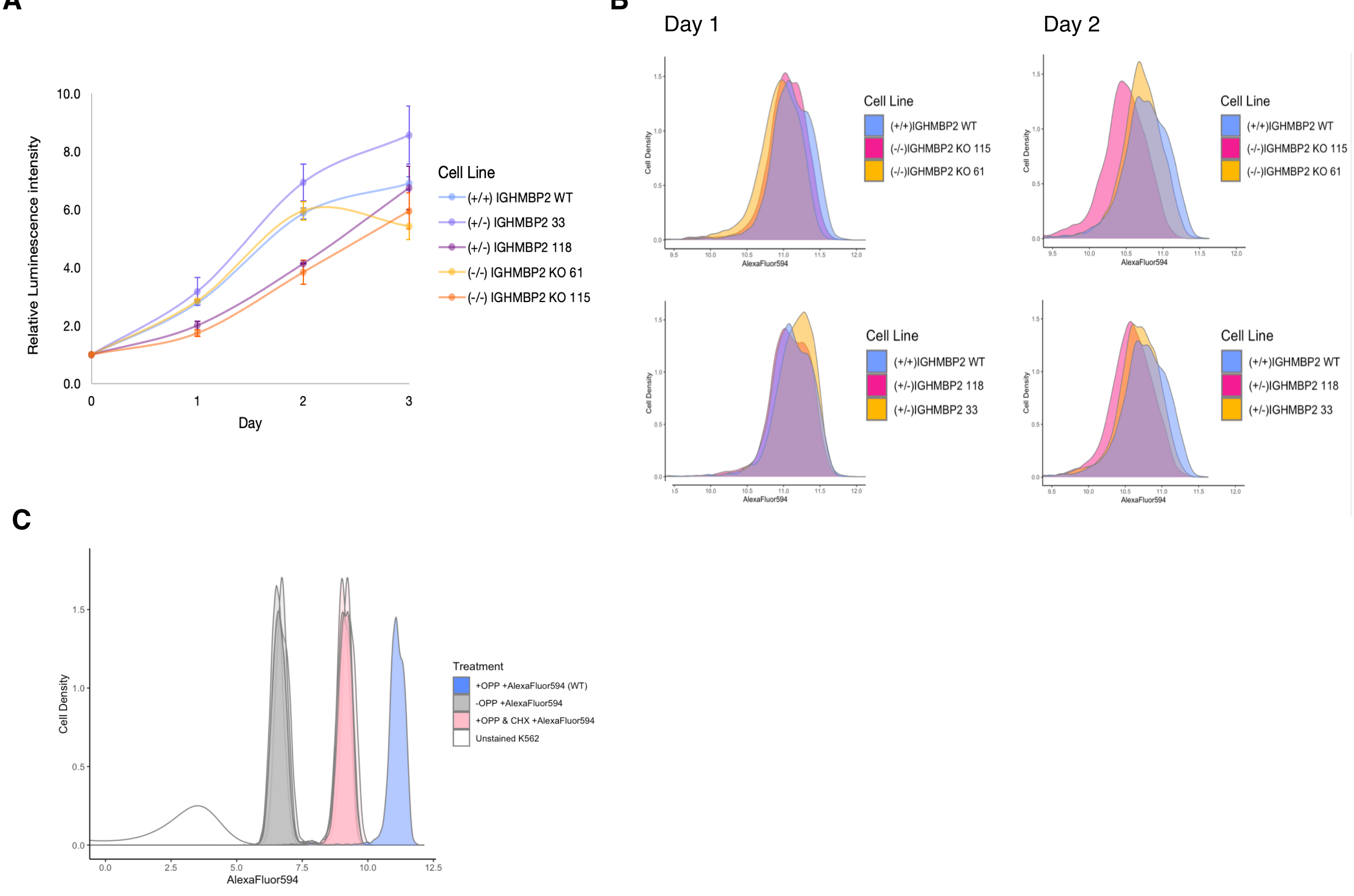

Figure S2. Nascent polypeptide synthesis assay with parental versus IGHMBP2 deletion clones.
(A) Growth rate profiles of K562 cell lines via CellTiter-Glo metabolic assay. Error bars are standard deviation between technical replicate luminescence readings. (B) Relative levels of global translation between K562 CRISPRi parental cells versus full or partial KO of IGHMBP2 as a function of AlexaFluor594 intensities via nascent protein synthesis assay, measured by flow cytometry. Cells were harvested at growth timepoints Day 1 and 2 reflected in *A*. (C) Representative OPP assay control results showing dynamic range between unstained, cells stained with AlexaFluor594 only without OPP, cycloheximide-treated cells, and +OPP+AlexaFluor594 cells treated with only DMSO.

bioRxiv preprint doi: https://doi.org/10.1101/2023.12.11.571166; this version posted December 12, 2023. The copyright holder for this preprint (which was not certified by peer review) is the author/funder, who has granted bioRxiv a license to display the preprint in perpetuity. It is made available under aCC-BY 4.0 International license.

Park_FigS3

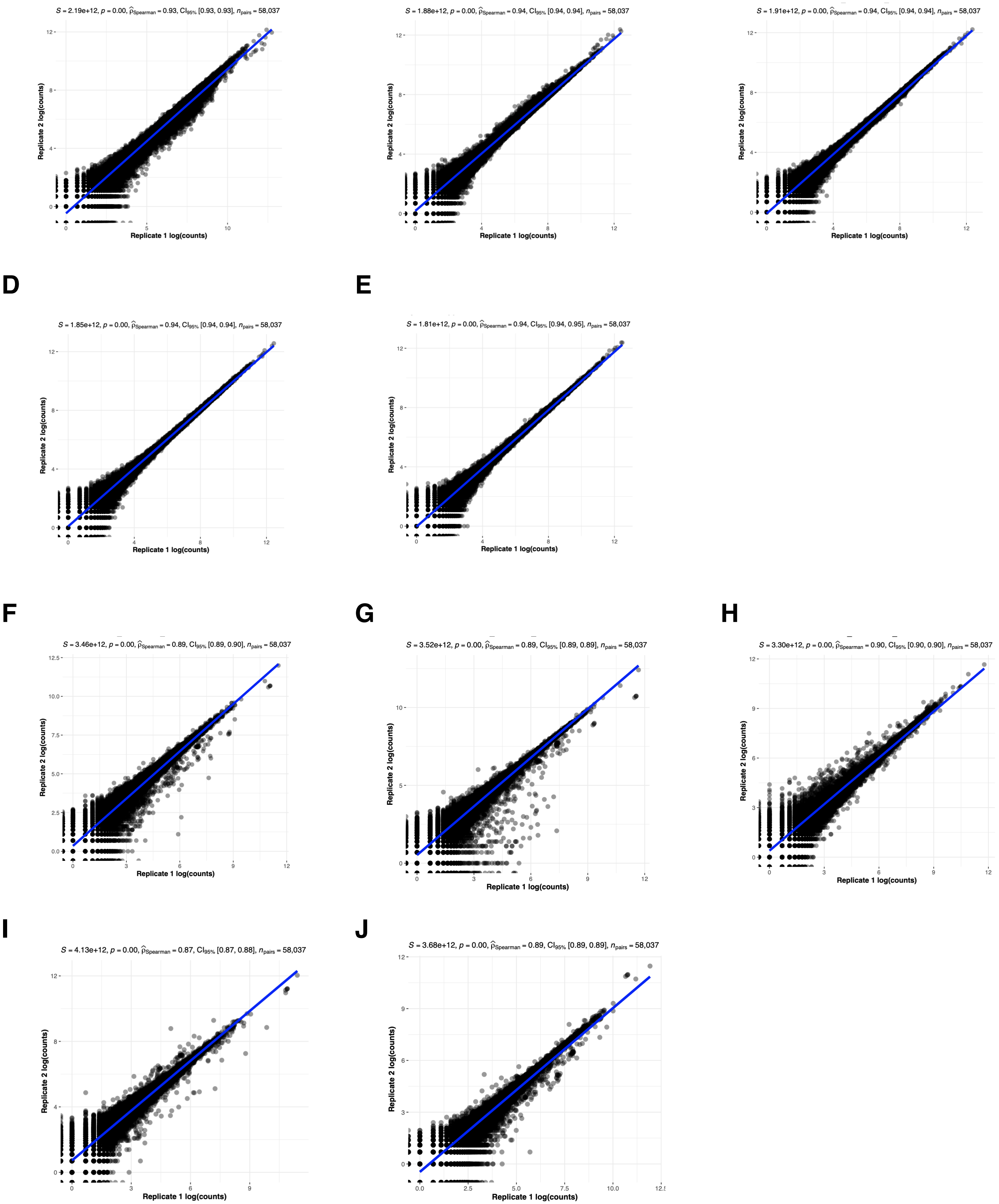

Figure S3. RNA-seq and Ribo-seq count correlation between replicate samples.
(A) RNA-seq Spearman's correlation analysis between parental, (B-C) HET Clones #1 and #2, and (D-E) KO Clones #1 and #2, respectively. (F) RNA-seq Spearman's correlation analysis between parental, (G-H) HET Clones #1 and #2, and (I-J) KO Clones #1 and #2, respectively.

bioRxiv preprint doi: https://doi.org/10.1101/2023.12.11.571166; this version posted December 12, 2023. The copyright holder for this preprint (which was not certified by peer review) is the author/funder, who has granted bioRxiv a license to display the preprint in perpetuity. It is made available under aCC-BY 4.0 International license.

Park_FigS4

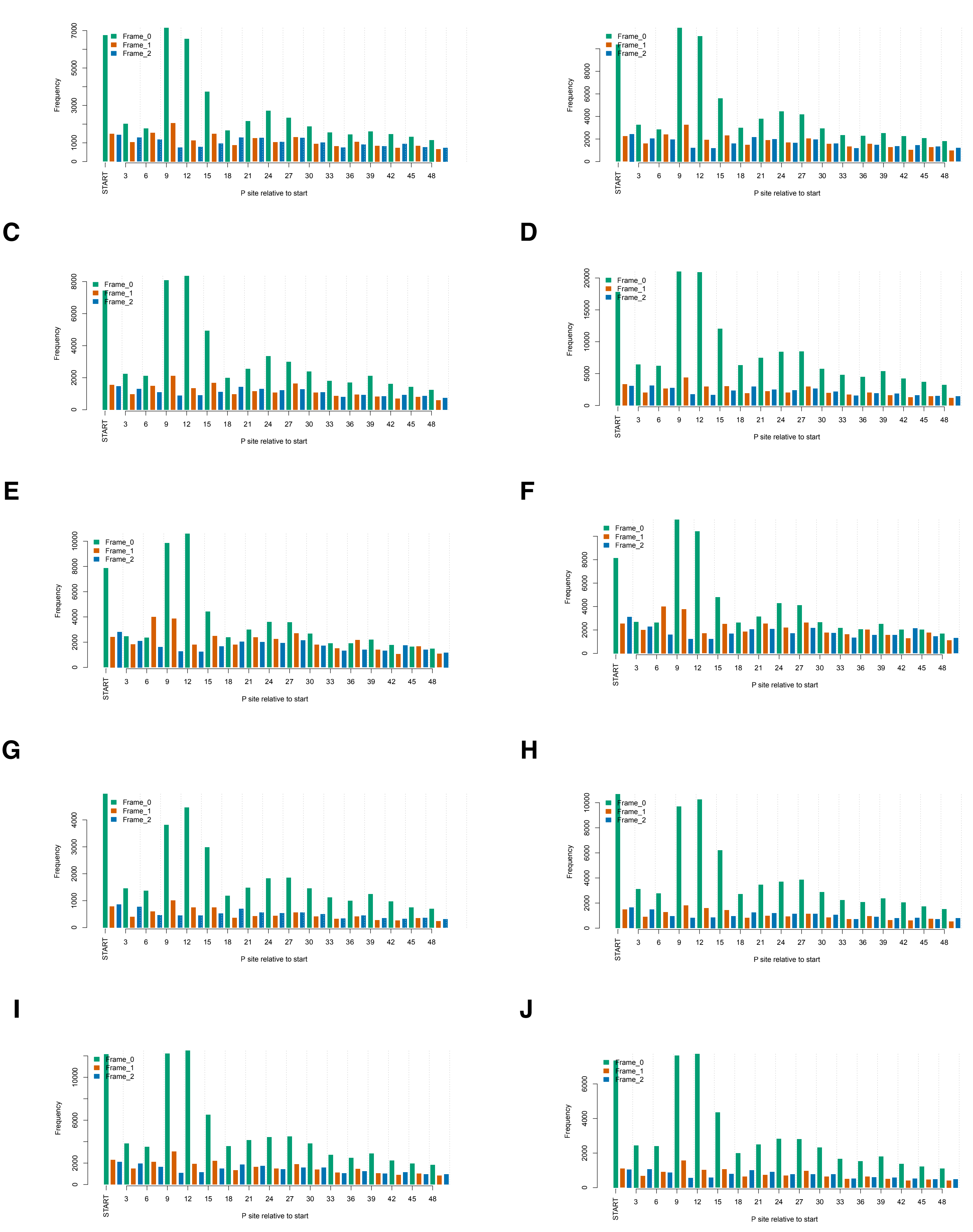

Figure S4. Periodicity profiles of Ribo-seq samples.
(A-J) 3-nt periodicity is shown for replicates 1 and 2 of Parental, HET Clone #1, HET Clone #2, KO Clone #1, and KO Clone #2, respectively.

bioRxiv preprint doi: https://doi.org/10.1101/2023.12.11.571166; this version posted December 12, 2023. The copyright holder for this preprint (which was not certified by peer review) is the author/funder, who has granted bioRxiv a license to display the preprint in perpetuity. It is made available under aCC-BY 4.0 International license.

Park_FigS5

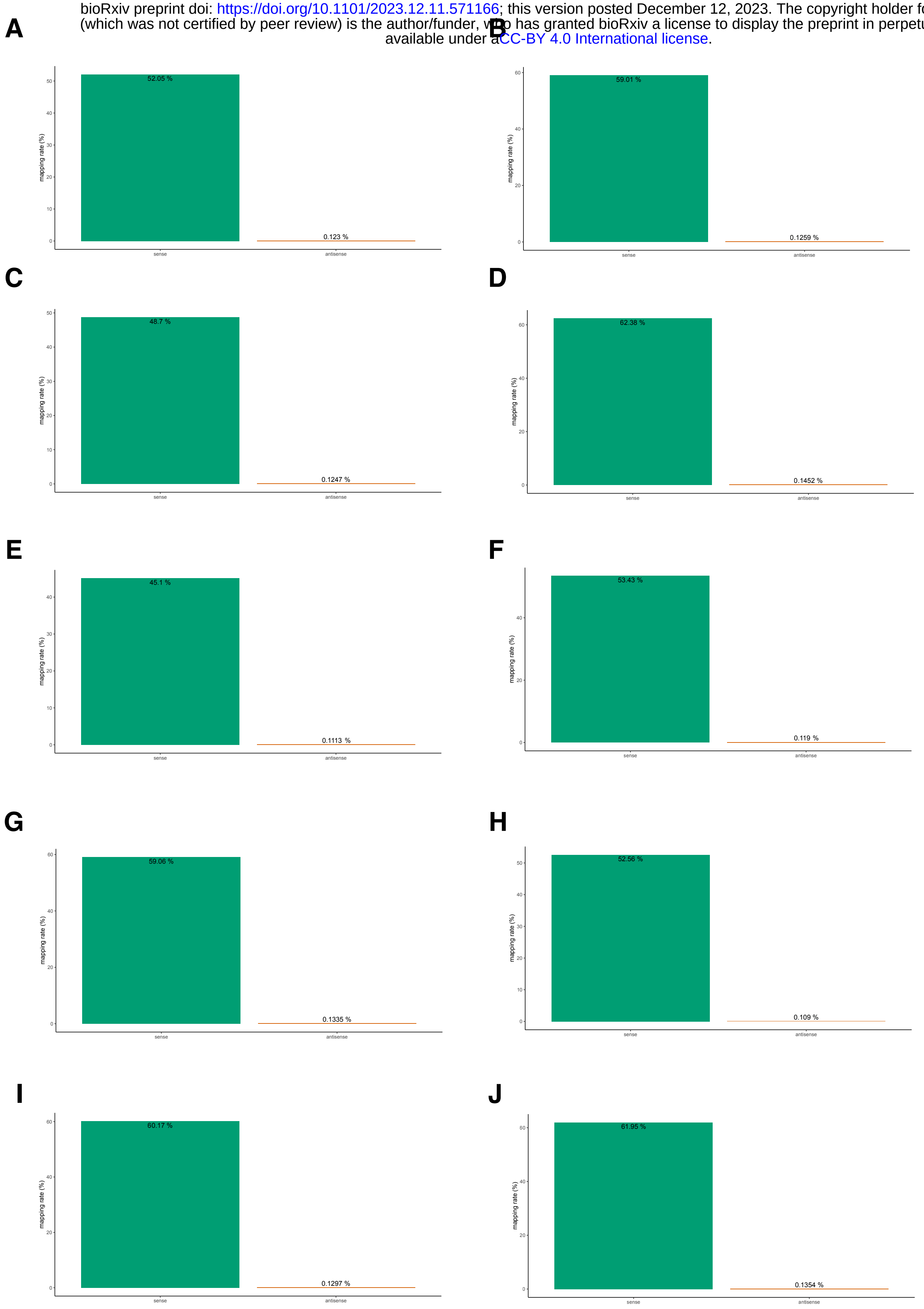

Figure S5. Strand sense analysis among Ribo-seq reads.
(A-J) Strand sense quantification is shown for replicates 1 and 2 of Parental, HET Clone #1, HET Clone #2, KO Clone #1, and KO Clone #2, respectively.

bioRxiv preprint doi: https://doi.org/10.1101/2023.12.11.571166; this version posted December 12, 2023. The copyright holder for this preprint (which was not certified by peer review) is the author/funder, who has granted bioRxiv a license to display the preprint in perpetuity. It is made available under aCC-BY 4.0 International license.

Park_FigS6

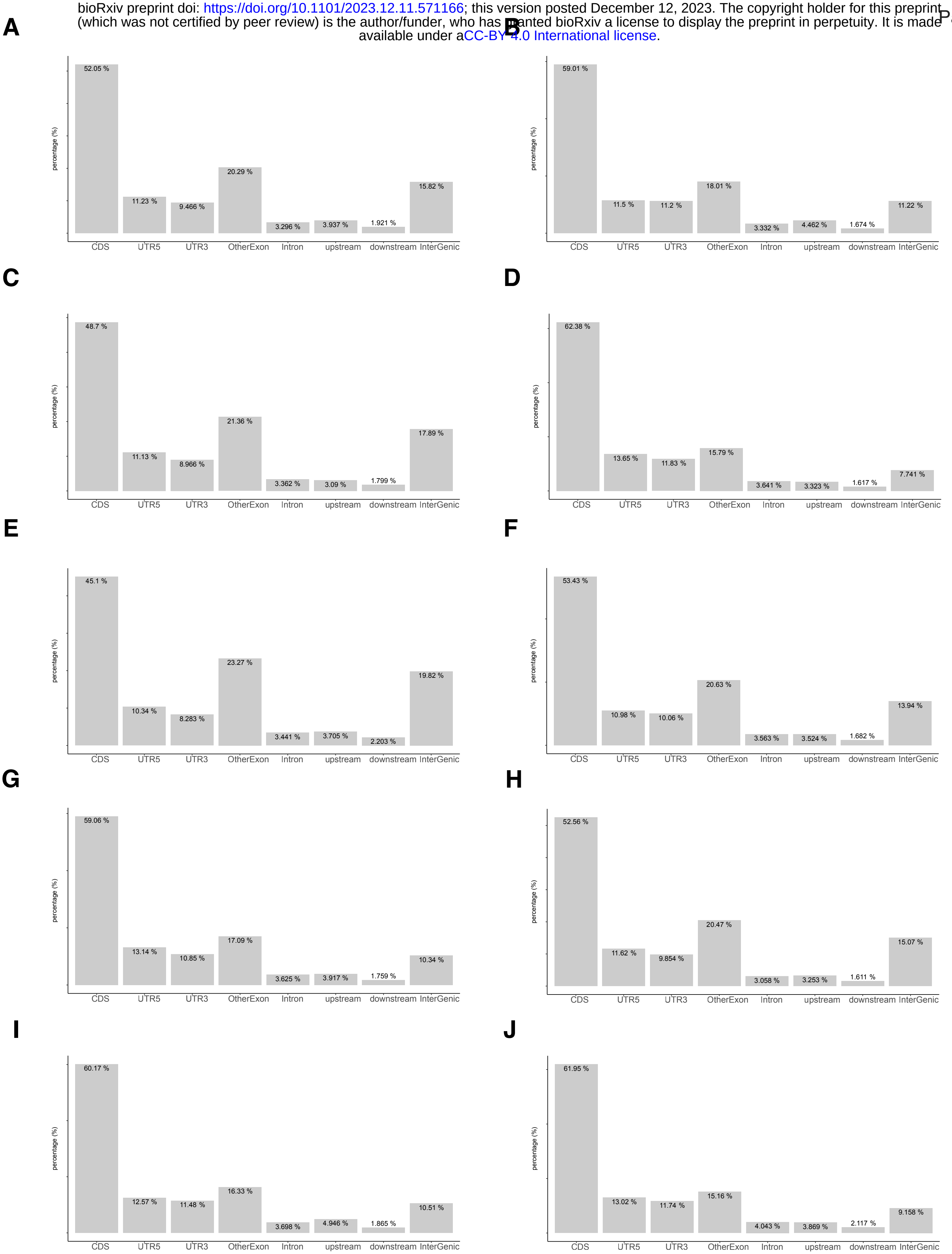

Figure S6. Read-mapping classifications of Ribo-seq samples.
(A-J) Read distributions among CDS, UTR, and other genomic classifications shown for replicates 1 and 2 of Parental, HET Clone #1, HET Clone #2, KO Clone #1, and KO Clone #2, respectively.

bioRxiv preprint doi: https://doi.org/10.1101/2023.12.11.571166; this version posted December 12, 2023. The copyright holder for this preprint (which was not certified by peer review) is the author/funder, who has granted bioRxiv a license to display the preprint in perpetuity. It is made available under aCC-BY 4.0 International license.

Park_FigS7

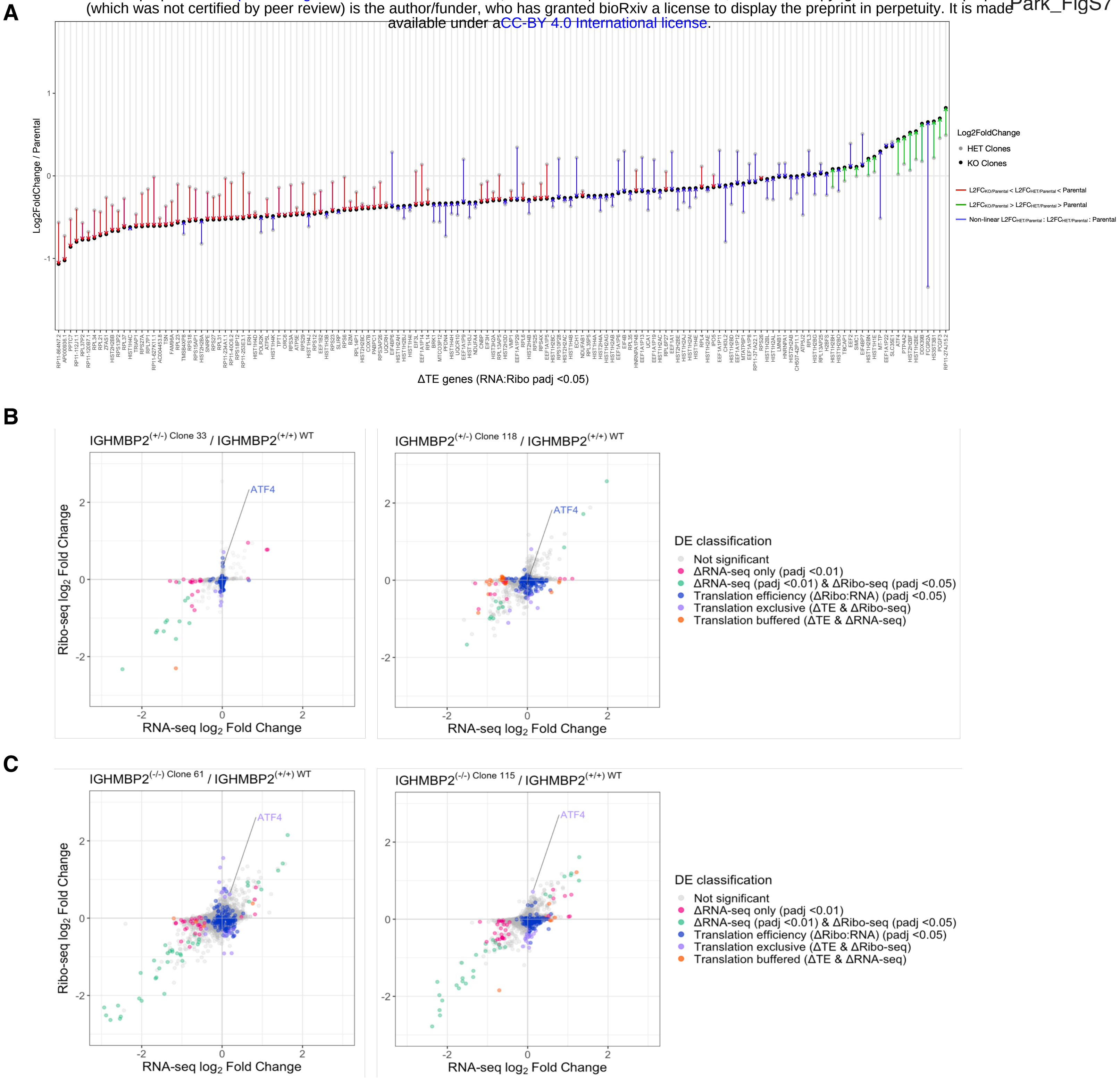

Figure S7. Translational efficiency of ATF4 is differentially upregulated in IGHMBP2 deletion cells.
(A) Average Log2foldchanges of TE DEGs in heterozygous and full deletion clones relative to parental cells. DEGs are sorted low to high average L2FC among KO clones. Average L2FC among HET clones were then plotted and connected with lines colored by relative directionality between KO, HET, and parental conditions, where linear changes are colored red or green for down and up-regulation with respect to parental, or blue for non-linear scenarios. (B) Ribosome profiling versus RNA-seq-derived shrunken log2 fold-changes per gene in clones with partial or (C) full IGHMBP2 deletion compared to parental cells. *ATF4* is highlighted, demonstrating translation-exclusive classification is gained in IGHMBP2 KO clones with reproducible Ribo:RNA-seq positioning trends. Differential expression (DE) analysis was performed using Wald test, and p-values were adjusted (p.adj) via Benjamini-Hochberg method. Cut-offs used for DE classifications are p.adj <0.01 for Δ RNA-seq (pink, green, and orange), and p.adj <0.05 for ΔRibo-seq (green & violet) and Δtranslation efficiency (TE; blue, violet, and orange). Genes with ΔTE across all clones were identified via likelihood ratio test against the Ribo:RNA-seq interaction term. Genes of both ΔTE and ΔRibo-seq are identified as translation exclusive (violet). Genes of ΔTE and Δ RNA-seq are classified as translation buffered (orange).

bioRxiv preprint doi: https://doi.org/10.1101/2023.12.11.571166; this version posted December 12, 2023. The copyright holder for this preprint (which was not certified by peer review) is the author/funder, who has granted bioRxiv a license to display the preprint in perpetuity. It is made available under aCC-BY 4.0 International license.

Park_FigS8

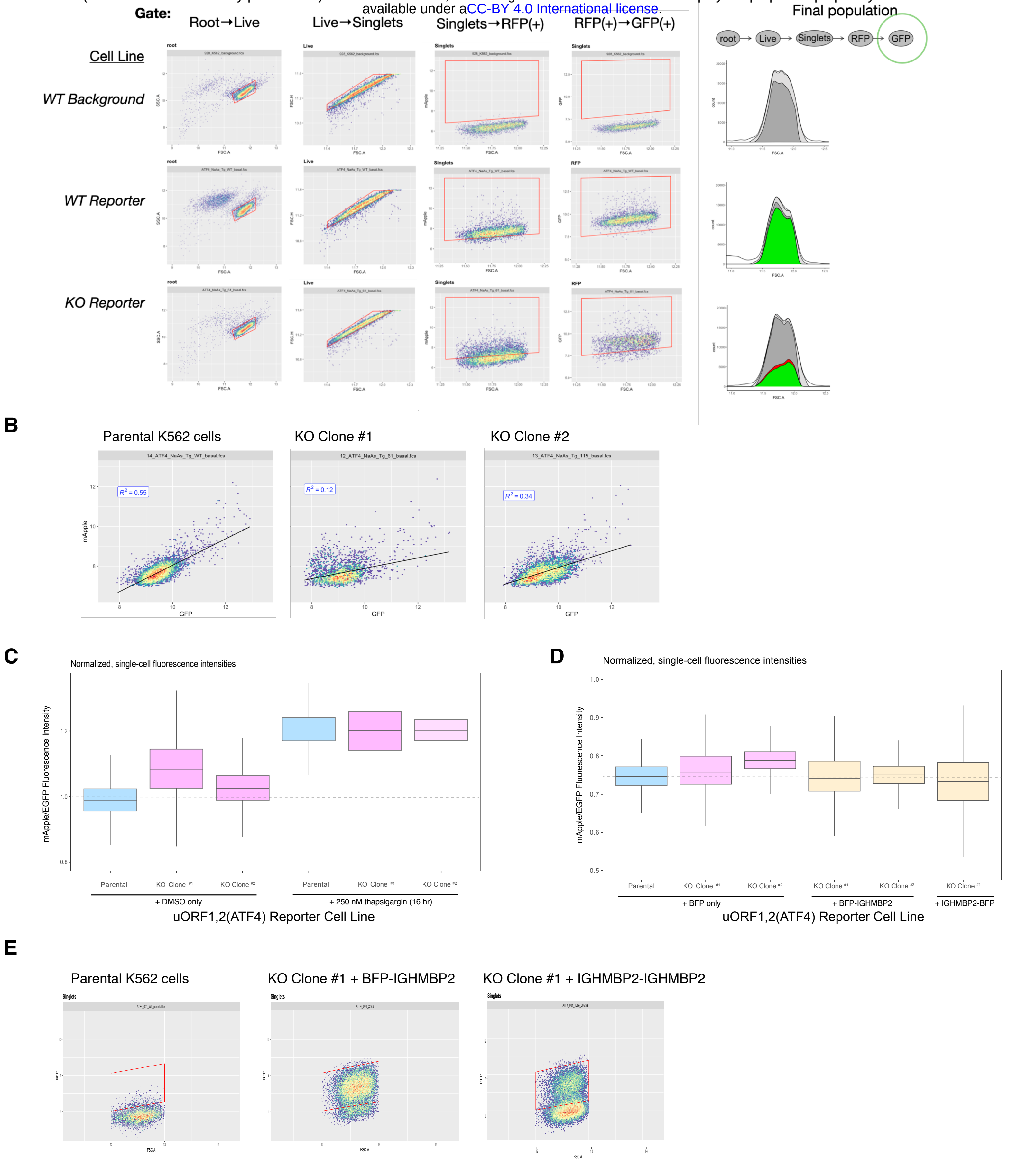

Figure S8. ATF4 reporter cell line characterization.
(A) Gating strategy for fluorescent signals of ATF4 reporter cell lines measured via flow cytometry. (B) Correlation between GFP and mApple expression at steady-state in cell lines stably expressing ATF4 reporter. (C) uORF1,2(*ATF4*)-mApple expression normalized to promoter and translational activity (mEGFP) in ΔIGHMBP2 K562 reporter cell lines at steady-state in DMSO or treated with 250 nM thapsigargin overnight. (D) Relative mApple/mEGFP intensities among reporter cell lines expressing BFP, BFP-IGHMBP2 or IGHMBP2-BFP, which were (E) gated for all BFP+ cells.

bioRxiv preprint doi: https://doi.org/10.1101/2023.12.11.571166; this version posted December 12, 2023. The copyright holder for this preprint (which was not certified by peer review) is the author/funder, who has granted bioRxiv a license to display the preprint in perpetuity. It is made available under aCC-BY 4.0 International license.

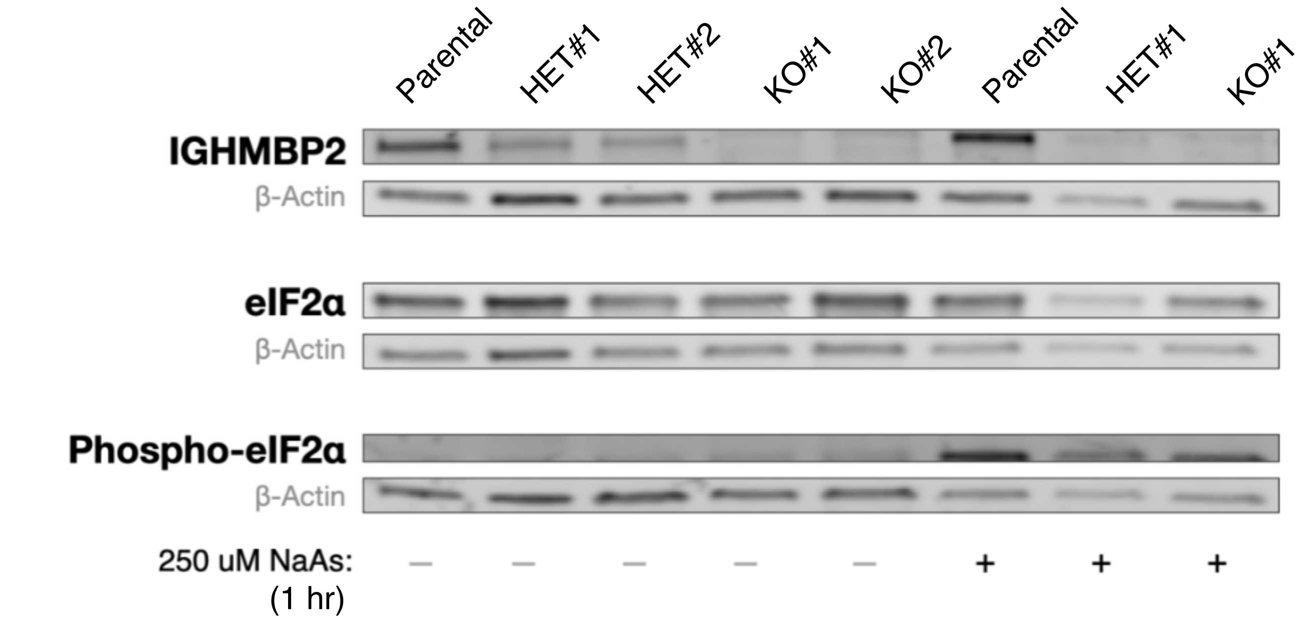

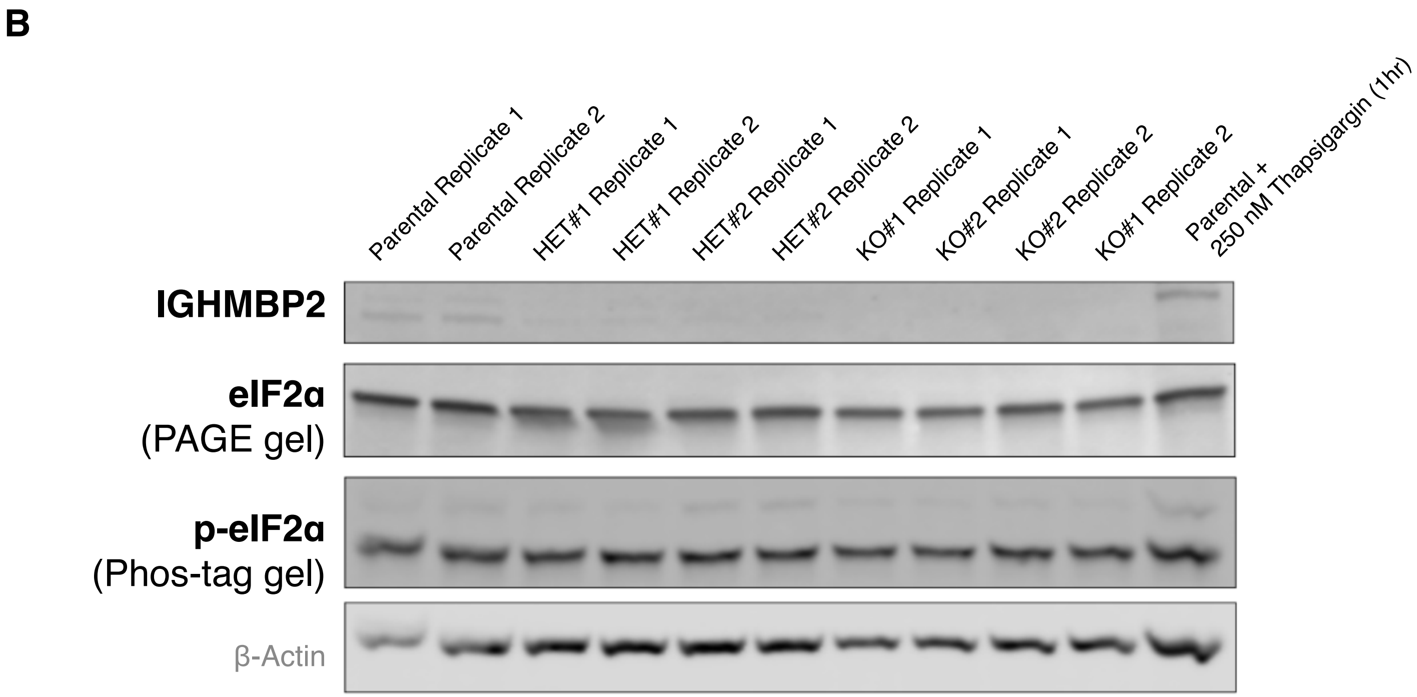

Figure S9. Differential p-eIF2α levels at steady-state in IGHMBP2 deletion clones is not detected by Western blot. (*A*) Western blots using PAGE with 50 µg total protein loaded per lane and (*B*) Phos-Tag gels loaded with 20 µg total protein from Parental, HET Clone #1, HET Clone #2, KO Clone #1, and KO Clone #2 cells. *B* also shows a corresponding control PAGE gel run in parallel to confirm p-eIF2α Phos-tag gel shifts are not attributed to degraded eIF2α. Blots shown in *A* and *B* were incubated with anti-eIF2α Ab, while a separate Western blot in A was incubated with anti-p-eIF2α(S51) Ab.

bioRxiv preprint doi: https://doi.org/10.1101/2023.12.11.571166; this version posted December 12, 2023. The copyright holder for this preprint (which was not certified by peer review) is the author/funder, who has granted bioRxiv a license to display the preprint in perpetuity. It is made available under aCC-BY 4.0 International license.

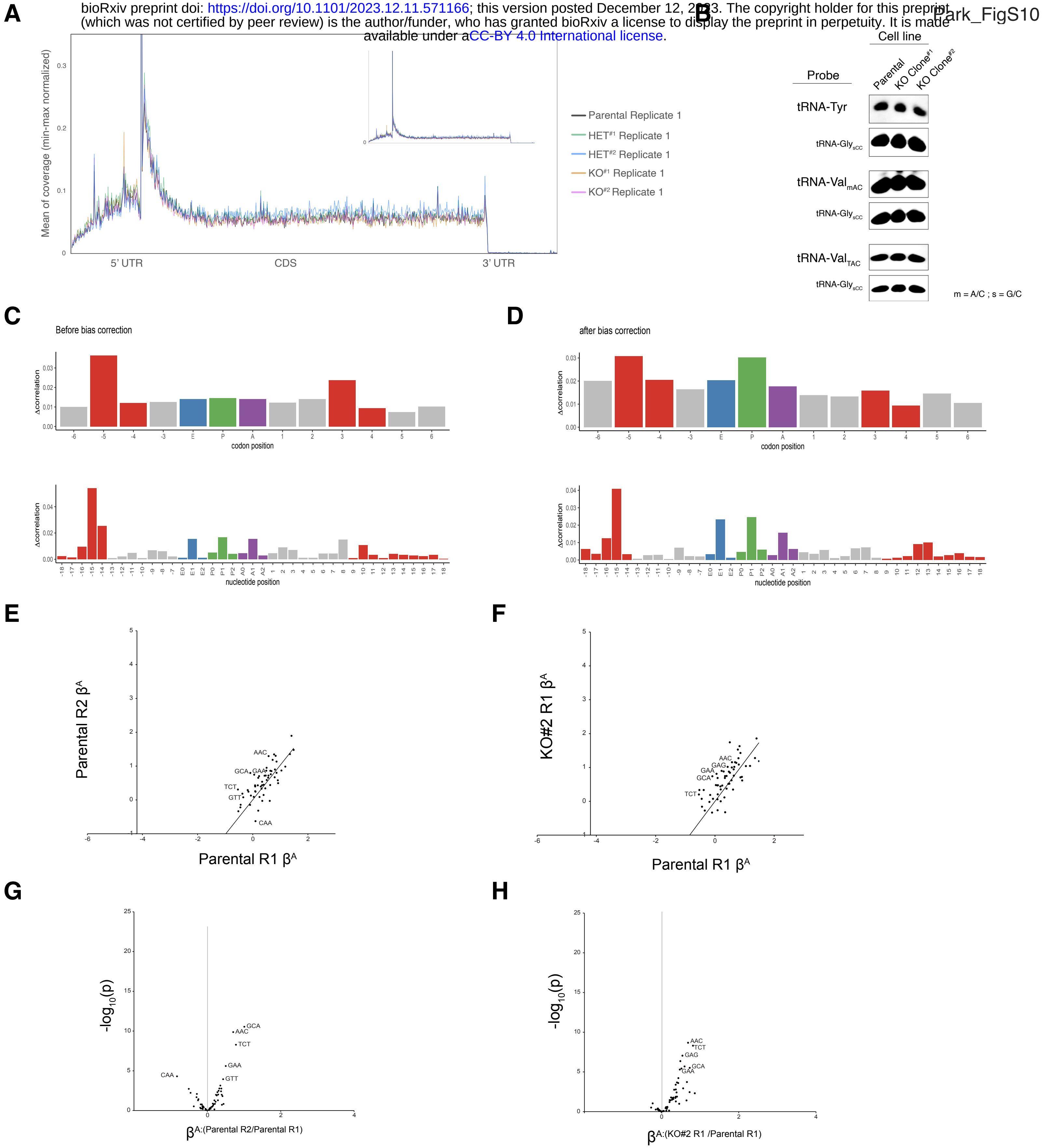

Figure S10. Ribosome footprint analyses across transcript regions and codons.
(*A*) Representative metagene analyses with Parental, HET Clone #1, HET Clone #2, KO Clone #1, and KO Clone #2 Ribo-seq data. Zoom-out view capturing the entire TSS peak is inset. (*B*) Northern blot visualizing tRNA-Tyr (all isodecoders), tRNA-Val$_{mAC}$ (GUU/C/G isodecoders), tRNA-Val$_{TAC}$ (GUA isodecoders), and tRNA-Gly$_{sCC}$ (GGC/U/G isodecoders) abundances in K562 cell lines with differential IGHMBP2 expression. 5 μg total RNA was loaded per lane. (*C-D*) Representative before and after footprint position bias-correction for Parental Ribo-seq sample processed with *choros*. (*E*) A-site codon regression coefficients between parental replicates and (*F*) KO Clone #2 relative to a parental sample. (*G*) Differential A-site codon enrichment visualized by regression coefficient interaction terms between parental replicates and (*H*) KO Clone #2 relative to a parental sample. *E* and *G* are shown to visualize expected noise among reference comparisons.

bioRxiv preprint doi: https://doi.org/10.1101/2023.12.11.571166; this version posted December 12, 2023. The copyright holder for this preprint (which was not certified by peer review) is the author/funder, who has granted bioRxiv a license to display the preprint in perpetuity. It is made available under aCC-BY 4.0 International license.

Park_FigS11

A

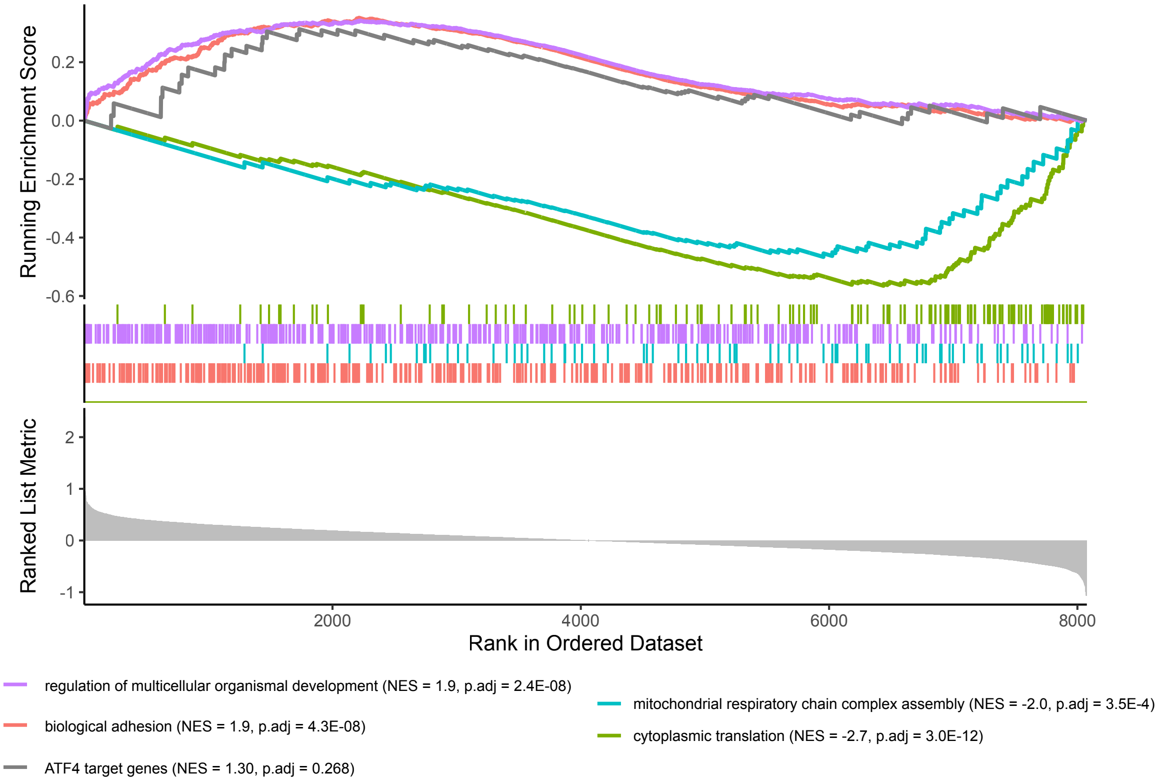

B

	log2FoldChange	p.adj
NLRP1	0.489	0.517
MALAT1	0.476	0.999
PPP1R15A	0.373	0.999
VARS	0.373	0.999
CLTCL1	0.369	0.999
KDM6B	0.346	0.999
FMNL1	0.343	0.999
GDF15	0.335	0.999
RHBDD2	0.301	0.999
DPYSL2	0.291	0.999
SESN2	0.291	0.999
BRF2	0.283	0.999
ASB1	0.268	0.999
PCK2	0.254	0.999
SLC3A2	0.253	0.999
TRIB3	0.249	0.999
MKNK2	0.221	0.999
CEBPB	0.218	0.999

C

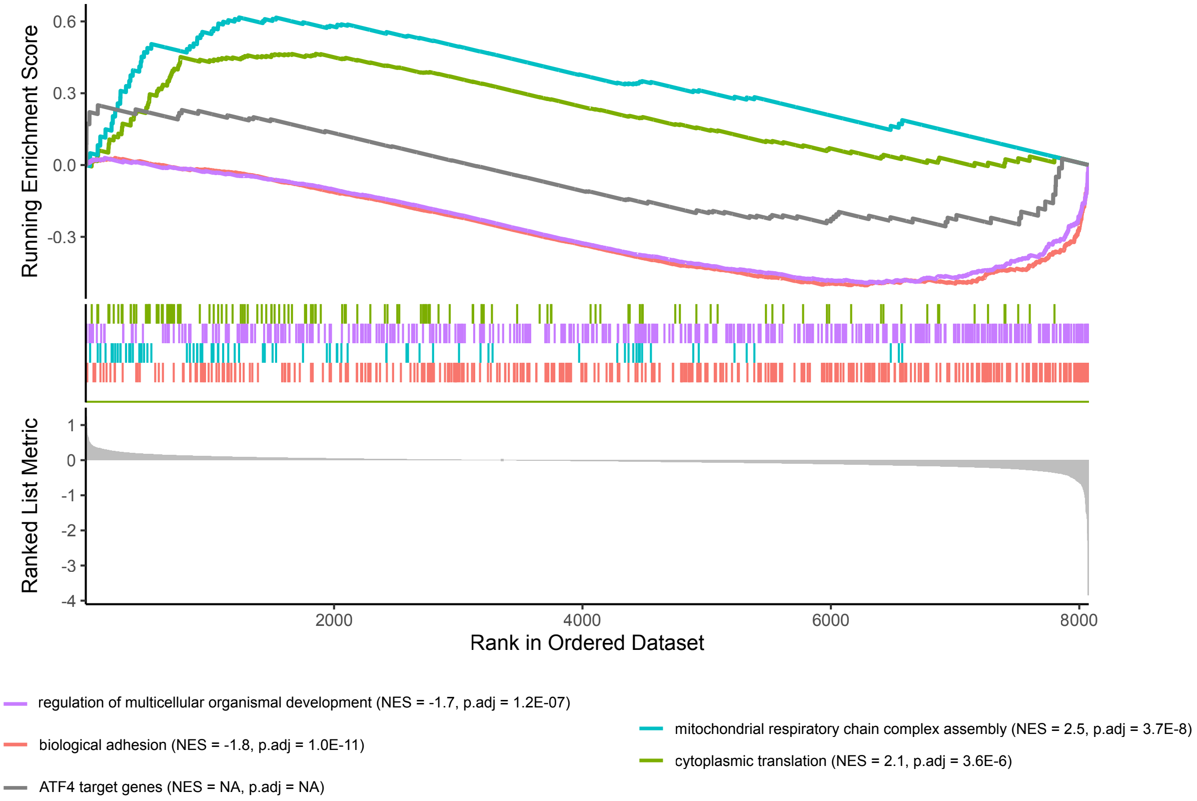

Figure S11. GSEA with ATF4 target gene list.
(A) Rank-gene profile of ATF4 target gene list among TE DEGs from IGHMBP2 KO clones, with representative significant gene sets shown for comparison. (B) Gene set list result from ATF4 target GSE in TE DEGs from IGHMBP2 KO clones, with average L2FC between KO clones #1 and #2 and p-adjusted values per gene shown. (C) Rank-gene profile of ATF4 target gene list among RNA-seq DEGs from IGHMBP2 KO clones, with representative significant gene sets shown for comparison.GSEA was computed using the Biological Processes ontology; min GS size = 25, max GS size = 1,000 with 100,000 permutations.
